# Effects of relaxation slide-films on the functional state of psychology students

**DOI:** 10.1192/j.eurpsy.2022.1790

**Published:** 2022-09-01

**Authors:** T. Zlokazova, A. Kuznetsova, M. Titova

**Affiliations:** Lomonosov Moscow State University, Department Of Psychology, Moscow, Russian Federation

**Keywords:** relaxation techniques, audio-visual slide-film, functional state

## Abstract

**Introduction:**

Visual images and music have long been known as a means to optimize emotions (Galeev, 1976; Gotsdiner, 1993). Together (eg in audio-visual slide-films) they may help students and professional cope with the negative side of intensive workloads (fatigue, stress, anxiety).

**Objectives:**

Our study attempts a multi-level assessment of the relaxation effect of a slide-film as part of student education.

**Methods:**

Sample - 46 psychology students. The 20 minute relaxation film was shown after lectures and discussion of a forthcoming examination. Measures: standard psychological and physiological functional state tests and a cognitive task before and after the film (Leonova & Kapitsa, 2003); an original questionnaire revealing individual associations and experiences felt during the film.

**Results:**

The results showed pronounced subjective discomfort and anxiety before the film. The relaxation film resulted in lowered blood pressure, increased subjective comfort, decreased fatigue and acute anxiety, and negative emotion scores, as well as higher productivity in performing the cognitive test (Student t-test, p<0.005-0.001). Concentration on the film subject, as well as the combination of the student’s thoughts and associations around the plot, showed a positive correlation with the amount of relaxation effect.

**Conclusions:**

Our research showed that using the relaxation slide-film (with imagery and music) can provide positive effects on students’ functional state. It also revealed the importance of the relevance of individual experience to the subject of the film to obtain optimal positive effects.

**Disclosure:**

No significant relationships.

